# Exploring the pathogenesis of insomnia and acupuncture intervention strategies based on the microbiota-gut-brain axis

**DOI:** 10.3389/fmicb.2024.1456848

**Published:** 2024-09-19

**Authors:** Jia Guo, Jixing Guo, Xiang Rao, Rongni Zhang, Qiang Li, Kun Zhang, Shanbo Ma, Jingyu Zhao, Changchun Ji

**Affiliations:** ^1^College of Acupuncture and Moxibustion, Shaanxi University of Chinese Medicine, Xianyang, China; ^2^Department of Acupuncture and Moxibustion, Shaanxi Hospital of Chinese Medicine, Xi’an, China; ^3^Department of Pharmacology, School of Pharmacy, Air Force Medical University, Xi’an, Shaanxi, China; ^4^Department of Pharmacy, Xijing Hospital, Air Force Medical University, Xi’an, Shaanxi, China; ^5^Department of Acupuncture and Moxibustion, Xi’an Hospital of Chinese Medicine, Xi’an, China

**Keywords:** microbiota-gut-brain axis, insomnia, pathogenesis, acupuncture intervention, strategies

## Abstract

Insomnia is a common sleep disorder observed in clinical settings, with a globally rising prevalence rate. It not only impairs sleep quality and daytime functioning but also contributes to a range of physiological and psychological conditions, often co-occurring with somatic and mental disorders. Currently, the pathophysiology of this condition is not fully understood. Treatment primarily involves symptomatic management with benzodiazepine receptor agonists, melatonin and its receptor agonists, sedative antidepressants, atypical antipsychotics, and orexin receptor antagonists. However, due to the adverse side effects of these drugs, including dependency, addiction, and tolerance, there is an urgent need for safer, more effective, and environmentally friendly treatment methods. In recent years, research on the microbiota-gut-brain axis has received significant attention and is expected to be key in uncovering the pathogenesis of insomnia. Acupuncture stimulates acupoints, activating the body’s intrinsic regulatory abilities and exerting multi-pathway, multi-target regulatory effects. A substantial body of evidence-based research indicates that acupuncture is effective in treating insomnia. However, the unclear mechanisms of its action have limited its further clinical application in insomnia treatment. Therefore, this study aims to elucidate the pathogenesis of insomnia from the perspective of the microbiota-gut-brain axis by examining metabolic, neuro-endocrine, autonomic nervous, and immune pathways. Additionally, this study discusses the comprehensive application of acupuncture in treating insomnia, aiming to provide new strategies for its treatment.

## 1 Introduction

Insomnia is characterized by subjective symptoms such as difficulty falling asleep or frequent awakenings with dreams, resulting in poor sleep quality, short sleep duration, and subsequent daytime fatigue, drowsiness, and lack of concentration. Epidemiological studies indicate that nearly 30% of the global population suffers from insomnia (Morin and Jarrin, 2022), with the prevalence rate in China reaching up to 45.5% (Chinese Society Of Neurology and Sleep Disorders Group, 2018). Chronic sleep deprivation can lead to reduced immunity, daytime lethargy, and fatigue, significantly affecting patients’ lives and work. In severe cases, it can cause a range of physiological and psychological conditions such as anxiety, depression, hypertension, diabetes, dementia, and cancer, imposing a substantial economic burden on society.

The pathophysiological mechanisms underlying insomnia have not been fully elucidated. Modern medicine attributes insomnia to abnormal neuronal activity in the brain, affecting neurotransmitters, hormone secretion, and the immune system. Primary clinical treatments for insomnia currently include benzodiazepines and medications based on melatonin. However, due to their significant side effects, including tolerance, dependency, and addiction (Zengjie et al., 2017), many patients find these treatments unacceptable. Acupuncture, an external therapy guided by principles of traditional Chinese medicine, lacks drug-related side effects. Acupuncture functions by stimulating acupoints to activate the body’s intrinsic regulatory abilities, exerting multi-pathway and multi-target regulatory effects. Evidence-based medical research (Kim et al., 2021) has demonstrated significant efficacy of acupuncture in treating insomnia. However, the mechanisms underlying its action continue to be a focus of investigation among scholars worldwide, thus limiting its broader clinical application in treating insomnia.

**Figure 1:**
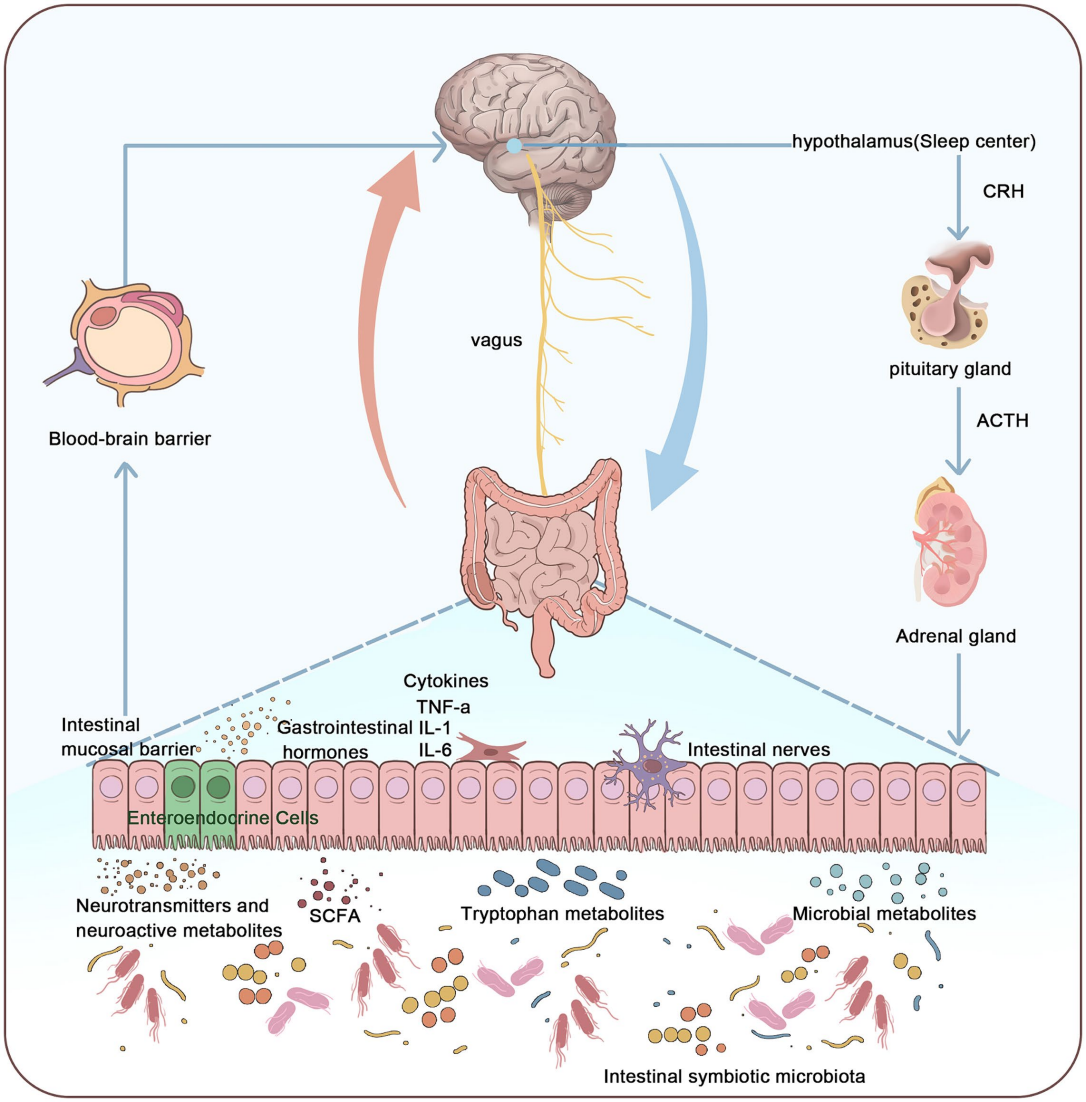
Relationship between the microbiota-gut-brain axis and the pathogenesis of insomnia. This figure illustrates that the pathogenesis of insomnia can be understood through the microbiota-gut-brain axis system. The microbiota-gut-brain axis connects the enteric nervous system (ENS) and the central nervous system (CNS), primarily influencing the CNS through the vagus nerve, gut microbiota, metabolites, neurotransmitters, and gastrointestinal hormones. This interaction activates the hypothalamic-pituitary-adrenal (HPA) axis, thereby affecting sleep. By integrating the gut microbiome, nervous system, endocrine system, and immune system, the microbiota-gut-brain axis forms a multidimensional, bidirectional regulatory pathway, thus maintaining the dynamic balance of the sleep–wake cycle.

The microbiota-gut-brain axis (MGBA) integrates the gut microbiota with the nervous, endocrine, and immune systems, establishing bidirectional regulatory pathways crucial for maintaining the dynamic balance of the sleep–wake cycle. This axis is closely linked to the pathogenesis of insomnia. However, existing studies often focus on mechanisms from individual perspectives, lacking a systematic approach. Therefore, this study aims to comprehensively understand insomnia from the perspective of the MGBA, elucidating metabolic, neuro-endocrine, autonomic nervous, and immune pathways. Additionally, this research explores the broader application of acupuncture in insomnia treatment, aiming to propose novel therapeutic strategies (Figure 1).

## 2 The microbiota-gut-brain axis

The MGBA refers to the bidirectional communication network between the gut microbiota and the central nervous system (CNS). This network is comprised of the nervous, endocrine, and immune systems, and includes several key components such as metabolic pathways, neuroendocrine regulatory pathways, the autonomic nervous system (ANS), and immune signaling. The following outlines the various aspects of how the MGBA influences the CNS.

The MGBA affects the CNS through metabolic pathways. Gut microbiota can impact host metabolism; for instance, neuroactive metabolites such as short-chain fatty acids (SCFAs) produced by the microbiota can influence neural development and function (Rothhammer et al., 2016). SCFAs produced by gut bacteria such as Clostridia and Bacteroides can cross the blood–brain barrier and directly enter the CNS (Onyszkiewicz et al., 2019), regulating the epigenetics of brain function genes and thus affecting CNS development. During early life, gut microbiota actively participates in the normal formation and functional construction of the nervous system. Gut-derived tryptophan metabolites can bind to aryl hydrocarbon receptors in the fetus through the placenta (Wei et al., 2021), significantly influencing fetal CNS development.

The MGBA influences the CNS through neuroendocrine regulatory pathways. Gut microbiota can alter host behavior and cognition by affecting the release of various neurotransmitters, including norepinephrine, serotonin (5-HT), and dopamine (Zhang et al., 2021), Additionally, gut microbiota can secrete various neurotransmitters such as gamma-aminobutyric acid (GABA), catecholamines, and histamine, which transmit signals to the CNS (Chen et al., 2021). These products can regulate neuronal activity in the brain through the MGBA, significantly impacting brain development and behavior. Furthermore, gut microbiota can influence brain function and emotional states by producing neurotransmitters and metabolic products, thereby regulating the activity of the hypothalamic-pituitary-adrenal (HPA) axis. Animal experiments have shown that early microbial colonization can affect the development and stress responses of the HPA axis in mice (Sudo et al., 2004). Additionally, certain probiotics may help reduce the release of stress hormones such as cortisol, thereby alleviating stress responses (Crumeyrolle-Arias et al., 2014).

The MGBA influences the CNS by regulating autonomic neural pathways. The gastrointestinal (GI) tract is densely innervated and regulated by the CNS, the ANS, and the enteric nervous system (ENS). Sensory information from the GI tract can be directly transmitted to the brain via the ENS or the vagus nerve (Doifode et al., 2020), Additionally, gut microbiota can influence the development and function of gut neurons by activating toll-like receptors (Doifode et al., 2020). Animal experiments have shown that electrical stimulation of vagal afferent fibers can alter the concentration of 5-HT in the brain, indicating that the brain and gut can communicate signals via the vagus nerve (Burgos et al., 2014). By stimulating the vagus nerve, gut microbiota can transmit gut inflammation signals to the CNS. Further studies have revealed that gut microbiota also regulate extrinsic sympathetic nerves in the gut. When the gut microbiota is lost, the expression of the neuronal transcription factor c-Fos in gut sympathetic ganglia is upregulated. Conversely, bacteria colonizing germ-free (GF) mice can inhibit c-Fos expression, thereby affecting the development and function of the CNS (Kuwahara et al., 2020).

The MGBA influences the CNS by regulating the immune system. The GI tract is the body’s largest immune system, with 70% of the lymphatic tissue attached to the intestinal mucosa. Immune cells in this lymphatic tissue play a protective role for the body. Cytokines produced by immune responses, such as interleukin-1 (IL-1) and tumor necrosis factor-alpha (TNF-α), circulate through the bloodstream to the hypothalamus, transmitting immune signals. These cytokines have potent immune functions, significantly impacting CNS activities.

In summary, the MGBA constructs a complex bidirectional communication network between the gut microbiota and the central nervous system through multiple mechanisms, including metabolic pathways, neuroendocrine regulation, autonomic nervous pathways, and the immune system. Through these pathways, gut microbiota can significantly influence the development, function, behavior, and emotional state of the central nervous system, revealing the critical role of gut microbes in neural health and disease.

## 3 The relationship between the microbiota-gut-brain axis and insomnia

Insomnia is closely linked to abnormalities in CNS function. The MGBA can regulate the CNS through various mechanisms, including metabolic, neuroendocrine, autonomic neural, and immune pathways, thereby potentially alleviating insomnia.

### 3.1 Metabolic pathways

The dysregulation of the MGBA can worsen CNS disorders by producing abnormal metabolites, thus affecting sleep. Examples include gut bacteria, SCFAs, and bile acids (BAs).

The genus Bacteroides is the largest Gram-negative bacterial group in the gut microbiota. Its production of lipopolysaccharides (LPS) and other endotoxins can activate the peripheral immune system, promoting infiltration of peripheral immune cells into the brain, thereby triggering CNS inflammation. According to reports (Szentirmai and Krueger, 2014), peripheral administration of LPS can increase the duration and intensity of slow-wave sleep while reducing paradoxical sleep.

Sleep deprivation may alter the composition of the gut microbiota, affecting the overall health and metabolic functions of the host. Specific probiotic strains can improve insomnia symptoms and may modulate physiological mechanisms related to sleep. Animal experiments (Wang et al., 2021), have found that chronic sleep deprivation in mice reduces the relative abundance of beneficial bacteria in the gut, suppressing the number of beneficial bacteria in feces nearly twofold. Other studies have shown that chronic insomnia patients have decreased levels of certain beneficial bacteria in the gut, such as bifidobacteria and lactobacilli, while potentially pathogenic bacteria like Enterobacteriaceae increase in number. The quantity of lactobacilli is negatively correlated with subjective sleep quality scores (Wang et al., 2021), suggesting that these microbial communities may play a role in sleep responses in humans. Another study (Chen et al., 2022a,b), using 16S rRNA gene sequencing of fecal samples from insomnia patients, revealed specific gut microbiota associated with reduced sleep efficiency, such as decreased levels of *Akkermansia muciniphila* correlating with lower sleep quality. In a randomized double-blind controlled trial (Chen et al., 2022a,b), insomnia patients treated with *Lactobacillus plantarum* PS128 probiotics showed significantly improved sleep quality, increased sleep efficiency, and longer sleep maintenance. Another study confirmed (Patterson et al., 2024) that intake of *Bifidobacterium infantis* 35624 probiotics had a positive impact on patients with irritable bowel syndrome and insomnia symptoms, improving their sleep quality and gut function.

Additionally, in the feces of insomnia patients, the concentration of SCFAs significantly decreases. SCFAs, derived from the microbial breakdown of carbohydrates such as acetate, propionate, and butyrate, are metabolic products produced by intestinal microbiota fermenting cellulose. They have been found to influence sleep through the gut-brain axis. Changes in the gut microbiota of insomnia patients may reduce the synthesis of SCFAs (Lehahn, 2022). Experiments have shown (Szentirmai et al., 2019 Silva et al., 2020) that butyrate can significantly increase non-rapid eye movement (NREM) sleep time in mice. SCFAs affect vagus nerve activity via the GPR43 receptor (Silva et al., 2020), thereby influencing reward and emotion regulation areas in the brain, which contributes to improving sleep.

BAs are among the most important metabolites of the intestinal microbiota, possessing multiple signaling functions. In a mouse model of insomnia, levels of BAs are generally elevated in both serum and ileum (Tianyue et al., 2023). Various BA receptors have been found in immune cells throughout the gastrointestinal tract. Evidence also suggests (Hang et al., 2019) that BAs have protective and anti-inflammatory effects on the brain. Therefore, activation of bile acid receptors in immune cells can trigger immune responses and modulate the CNS.

### 3.2 Neuroendocrine pathways

The neuroendocrine regulatory pathway is a crucial mechanism of the MGBA, influencing the CNS by adjusting levels of neurotransmitters and brain-gut peptides, thereby affecting sleep.

Brain-gut peptides (BGPs) are a significant class of gastrointestinal hormones in the neuroendocrine signaling pathway, exerting dual actions as hormones and neurotransmitters. They are widely distributed in gastrointestinal and brain tissues. Certain BGPs such as serotonin (5-HT), melatonin (MT), substance P (SP), and gamma-aminobutyric acid (GABA) play regulatory roles in sleep.

5-HT, an important neurotransmitter in the gastrointestinal tract, accounts for 95% of the total body serotonin produced in the gut (Jones et al., 2020). It is considered a crucial biological substrate in the pathogenesis of mood disorders. Besides, 5-HT plays a significant role in regulating the structural integrity of the enteric nervous system (ENS), immune responses, and epithelial cell integrity. 5-HT initiates and sustains slow-wave sleep to facilitate sleep regulation (Rancillac, 2016). During wakefulness, 5-HT is released from nerve terminal axons, influencing the synthesis of specific sleep-related substances in the brain. Gut microbiota stimulate 5-HT production through TpH1 activation (Legan et al., 2022), and critical metabolites such as SCFAs and secondary bile acids also impact 5-HT synthesis.

Gastrointestinal hormones are distributed in gastrointestinal tissues and transmit endocrine-neural impulse signals bidirectionally through the blood–brain barrier. Current research indicates that the mechanism of gastrointestinal hormones regulating central nervous signals may be related to the activation of G protein-coupled receptors. SCFAs and BAs can activate the G protein-coupled bile acid receptor TGR5, thereby stimulating the release of 5-HT (Chen et al., 2022a,b). Additionally, MT derived from 5-HT can regulate the body’s circadian rhythm and sleep cycle (Pandi-Perumal et al., 2008), and it also inhibits gastric acid secretion, protects the gastric mucosa, and promotes gastrointestinal motility.

MT is a hormone secreted by the pineal gland in the brain, playing a crucial role in regulating the human circadian rhythm and sleep patterns. MT can influence the sleep–wake cycle through its receptors MT1 and MT2 (Rashid et al., 2024), and it is involved in regulating sleep structure and sleep quality. The gut microbiota can indirectly affect the synthesis and secretion of MT through the gut-brain axis. Some metabolic products produced by gut bacteria, such as SCFAs, have been shown to influence MT production. Additionally, dysbiosis of the gut microbiota can affect MT synthesis, thereby impacting sleep. One study demonstrated (Wang et al., 2023) that transplanting the gut microbiota from sleep-deprived mice to normal mice resulted in disrupted MT synthesis and secretion in the recipient mice, leading to decreased sleep quality.

Additionally, substance P (SP) is an excitatory neurotransmitter composed of 11 amino acids. SP can alter sleep or slow-wave sleep activity through its receptors. Studies have shown (Asarnow, 2019) that injecting SP into the lateral ventricle of rats can increase sleep time, categorizing it as a central sleep-promoting substance. It can regulate the expression levels of various substances in brain tissue and promote sleep in the CNS by exciting neurons in the hypothalamic preoptic area (Yanlong, 2004). Furthermore, chronic sleep deprivation can lead to reduced microbial adhesion and penetration in the distal ileum and cecum, elevated serum cytokine levels (Poroyko et al., 2016), and decreased pro-adrenaline levels, with these changes worsening with prolonged sleep deprivation.

GABA is an inhibitory neurotransmitter that plays important physiological roles in humans, including reducing neuronal activity, regulating heart rate, enhancing memory, and modulating hormone secretion. Studies have shown (Yu et al., 2020) that GABA-fermented milk can improve sleep, with its protective mechanism possibly involving the regulation of gut microbiota and increased SCFA levels. Experiments have confirmed (Strandwitz et al., 2019) that GABA serves as a growth factor for certain bacteria, thereby regulating the gut microbiota. Another study indicated (Mabunga et al., 2015) that GABA extracted from fermented rice germ can counteract caffeine-induced sleep disorders without affecting the general activity of mice.

Additionally, gut microbiota can influence sleep activities by regulating the hypothalamic-pituitary-adrenal (HPA) axis. High levels of HPA axis activity can cause sleep disorders, and correspondingly, sleep disturbances can further dysregulate HPA axis function (Qinghai and Rongyu, 2006). Sleep disorders may be associated with overactivation of the HPA axis (Manosso et al., 2024). Gut microbiota can affect HPA axis levels (Ge et al., 2021), and supplementing with probiotics can regulate HPA axis activity, thereby improving sleep quality (Lan et al., 2023).

### 3.3 Autonomic nervous pathways

The autonomic nervous system (ANS) includes the sympathetic nervous system, parasympathetic nervous system, and enteric nervous system (ENS), with the primary component of the parasympathetic nervous system—the vagus nerve—playing an important role in regulating sleep and emotions due to its extensive distribution and functions. The vagus nerve pathway is a major route through which the gut microbiota influences the CNS, as activating the vagus nerve has significant anti-inflammatory effects that help mitigate excessive inflammatory responses triggered by the gut microbiota. This neural pathway acts as a bridge between the gut and the CNS. Vagal afferent fibers regulate sleep by translating inflammatory signals between the brain and the periphery. These fibers can transmit inflammatory stimuli (Zielinski and Gibbons, 2022), from peripheral viscera to stimulate the nucleus of the solitary tract (NTS) in the brainstem. The NTS projects to multiple sleep-regulating brain areas, which can induce the expression of pro-inflammatory sleep-inducing molecules and effects. The efferent fibers of the vagus nerve, partly mediated by acetylcholine neurotransmitters in the dorsal motor nucleus (DMN) and the nucleus ambiguus (NA), have anti-inflammatory effects and can reduce the release of peripheral inflammatory factors. The suprachiasmatic nucleus (SCN) can play a role in regulating the circadian rhythms of brain and peripheral molecules. Studies have confirmed (Morais et al., 2020) that *Bifidobacterium longum* may exert anxiolytic effects by signaling to the CNS through the vagus nerve pathway at the ENS level. Hansen et al. (1997) demonstrated through animal experiments that the subdiaphragmatic vagus nerve plays a crucial role in conveying sleep and fever signals to the brain. Yue Jiao and colleagues (Jiao et al., 2020) confirmed through clinical trials that transcutaneous vagus nerve stimulation might be an effective option for treating insomnia. Additionally, vagus nerve stimulation can regulate sleep by altering neurotransmitter levels, and GABA produced by the gut microbiota may influence CNS functions through the vagus nerve (Lucena et al., 2021). Studies have shown (Xian and Aihua, 2015), that stimulating the main trunk or superficial branches (such as the auricular branch) of the vagus nerve increases afferent impulses, which can elevate CNS levels of neurotransmitters like GABA and reduce concentrations of excitatory neurotransmitters like glutamate, thereby improving nighttime sleep quality, reducing daytime sleepiness, and enhancing mood. This process plays a crucial role in sleep regulation, further demonstrating the close link between gut microbiota and sleep quality.

### 3.4 Immune pathway

The immune regulation pathway is a critical mechanism of the MGBA. The gut microbiota interacts with intestinal epithelial cells, regulates the function of the intestinal barrier, and influences the maturation and activation of immune cells, thereby affecting brain behavior and function.

Gastrointestinal bacteria have been proven to play crucial roles in the initiation, control, regulation, and implementation of the immune system (Belkaid and Hand, 2014). Gastrointestinal bacteria can induce intestinal T cells to migrate to the brain (Zhou et al., 2022). These T cells residing in the brain can regulate the transition of microglia from immature to mature states. In addition to participating in immune functions within the CNS, microglia are also involved in various stages of neuronal activity, including synaptic remodeling to improve neural network connectivity. Researchers have found (Mossad and Erny, 2020) that the lack of a complex host microbiota in the gastrointestinal tract increases the number of microglia in the CNS and leads to defects in microglia maturation, activation, and differentiation, ultimately resulting in impaired immunity to bacterial or viral infections. Additionally, there is growing evidence (Erny et al., 2015) that SCFAs have various impacts on microglial function. Studies have shown that supplementing GF mice with SCFAs can reduce the production of abnormal microglia. Li et al. (2023) demonstrated through animal experiments the critical role of SCFAs in maintaining microglial homeostasis against neuroinflammatory stimuli. Metabolites produced by the gut microbiota, such as SCFAs, can cross the blood–brain barrier, affecting microglia and the synthesis and release of neurotransmitters (Van de Wouw et al., 2018), thereby regulating emotions and behavior. Some researchers believe (Fock and Parnova, 2023) that SCFAs can influence blood–brain barrier permeability, thereby regulating the brain’s sensitivity to other sleep-regulating factors.

Approximately 70% of immune cells are located in the gut-associated lymphoid tissue, where they can release various cytokines including interleukins and tumor necrosis factor (TNF). Molecules derived from the gut microbiota may act as antigens in the brain. Several members of the interleukin-1 (IL-1) family have been shown to regulate sleep (Zielinski et al., 2016), including IL-1β, IL-1α, IL-18, and IL-37. The sleep-modulating effects of these IL-1 family members partly depend on the activation of downstream receptors. Researchers suggest (Ingiosi et al., 2015) that increased levels of IL-1 in the brain significantly prolong non-rapid eye movement (NREM) sleep, while reducing IL-1 secretion decreases total sleep time, demonstrating the influence of IL-1 levels on sleep duration. Other studies have found (Krueger et al., 2011) that bacteria can promote sleep by enhancing cytokines such as TNF and IL-1, which act on individual neuronal circuits and sleep-regulating centers in the brain, influencing sleep through substances like nitric oxide, adenosine, and glutamate. Animal experiments have shown (Fernandes, 2006), that TNF-α transiently inhibits the gene expression of melatonin precursors Aa-nat, hiomt, and N-acetyl-5-hydroxytryptamine in the pineal gland of rats, indicating that the nocturnal surge of melatonin is impaired at the onset of inflammation and recovers during acute response shutdown or chronic inflammation. Furthermore, probiotics such as bifidobacteria, lactobacilli, and *Escherichia coli* can affect the levels of inflammatory factors in the blood (Cristofori et al., 2021), and levels of inflammatory factors like IL-1, interleukin-6 (IL-6), and TNF are significantly elevated in patients with sleep disorders, suggesting a potential link between these microbial communities and immune pathways regulating sleep.

## 4 Acupuncture intervention strategies

Traditional Chinese Medicine (TCM) places great emphasis on the relationship between the brain and the gastrointestinal system when treating insomnia. The ancient theory that “if the stomach is not in harmony, sleep will be disturbed” aligns with the modern concept of brain-gut interaction, providing an objective basis for the holistic approach of TCM diagnosis and treatment. TCM offers various methods for treating insomnia, among which acupuncture stands out as a green and effective treatment option, demonstrating unique advantages. Acupuncture methods for treating insomnia include body acupuncture, scalp acupuncture, abdominal acupuncture, auricular acupuncture, moxibustion, and other techniques. By stimulating specific acupoints, acupuncture can regulate the body’s intrinsic balance and exert a comprehensive regulatory effect.

### 4.1 The efficacy of acupuncture in treating insomnia and related research

Acupuncture treatment for insomnia has proven effective in clinical practice. Numerous clinical studies and systematic reviews have shown that acupuncture can significantly improve sleep quality in patients with insomnia, shorten sleep onset time, extend sleep duration, reduce the number of nighttime awakenings, enhance sleep efficiency, and decrease daytime sleepiness and anxiety-depressive symptoms. Yichuang (2023) found through a meta-analysis that acupuncture treatment for insomnia is effective and superior to non-acupuncture treatments, with stable results, providing scientific evidence for the efficacy of acupuncture in treating insomnia. A randomized controlled trial (Guo et al., 2013) divided patients into acupuncture, estazolam, and sham acupuncture groups, and found that all groups showed improvement compared to baseline, with the acupuncture group demonstrating significantly better sleep quality and vitality, and reduced daytime dysfunction and sleepiness compared to both the pharmaceutical and sham acupuncture groups. Another study (Qiang et al., 2020) discovered that acupuncture has similar or even better effects compared to medication in improving sleep quality in insomnia patients, and that acupuncture treatment has a longer-lasting effect and lower relapse rate (Xu et al., 2024). Additionally, neuroimaging studies (Zang et al., 2023) have found that acupuncture can regulate brain functional connectivity in insomnia patients and affect brain activity related to sleep, thereby improving insomnia. This indicates that acupuncture not only alleviates insomnia symptoms but also offers stable long-term effects, ultimately enhancing patients’ quality of life.

### 4.2 Acupuncture’s regulatory effect on the microbiota-gut-brain axis

Acupuncture, as a holistic regulatory method, stimulates specific acupoints to influence the body’s nervous, endocrine, immune, and other systems, thereby regulating the function of the MGBA to treat insomnia.

Research indicates (Hao et al., 2022), that acupuncture can improve intestinal peristalsis and secretion, enhance intestinal mucosal barrier function, and regulate intestinal immune responses, thereby modulating the structure and quantity of gut microbiota. Xu et al. (2023) confirmed through animal experiments that electroacupuncture can significantly increase the production of butyrate and regulate gut microbiota to promote intestinal peristalsis. Qiu et al. (2023) demonstrated that electroacupuncture can exert antidepressant effects by modulating the abundance of lactobacilli and staphylococci. Additionally, acupuncture can regulate metabolic products produced by the gut microbiota, such as SCFAs and bile acids. A clinical trial (Bao et al., 2022) showed that acupuncture treatment three times a week for 12 weeks can increase the ratio of SCFAs and anti-inflammatory bacteria, and effectively reduce the levels of LPS and Th1/Th17-related cytokines in the blood, thereby enhancing intestinal barrier function. These studies suggest that acupuncture may help restore the balance of the gut microbiota and treat insomnia by influencing the metabolic pathways of the MGBA.

In recent years, several studies have found that acupuncture can regulate neurotransmitters and cytokines, thereby improving sleep. Research shows (Provided et al., 2019) that acupuncture can promote the release of neurotransmitters such as 5-HT, GABA, and MT in the brain, thereby regulating neuronal activity and improving insomnia symptoms. Another study (Chen et al., 2023) indicated that depressive-like behaviors in rats significantly improved after 4 weeks of acupuncture treatment, with increased expression of 5-HT. Jinpeng et al. (2022) used retained needle therapy at the Five Heart Points combined with repetitive transcranial magnetic stimulation to treat post-stroke insomnia. The results showed that after 4 weeks of treatment, patients had significantly elevated serum GABA and 5-HT levels compared to before treatment, along with improved sleep quality and reduced anxiety and depression. Hong et al. (2020) confirmed that melatonin levels in the pineal gland of mice receiving acupuncture treatment significantly increased, thereby improving sleep outcomes. Furthermore, acupuncture can regulate the neuroendocrine axis (Yi et al., 2023), reducing levels of stress hormones such as adrenaline and cortisol, thus alleviating sleep disorders. These studies suggest that acupuncture may influence the neuroendocrine pathways of the MGBA by modulating neurotransmitters and cytokines, thereby treating insomnia.

Stimulation of specific acupuncture points can also activate the vagus nerve (Li et al., 2022), thereby balancing the activity of the sympathetic and parasympathetic nervous systems, reducing the body’s stress response, promoting physical and mental relaxation, and improving sleep onset and quality.

The research conducted by Liu et al. (2020) suggests that auricular acupuncture can alleviate depressive symptoms, such as anxiety, cognitive impairment, and sleep disturbances, by activating the vagus nerve. Rong et al. (2016) confirmed through clinical trials that transcutaneous auricular vagus nerve stimulation can significantly improve depressive symptoms in patients. These studies indicate that acupuncture may treat insomnia by stimulating the vagus nerve, thereby affecting the autonomic nervous pathways of the MGBA.

By stimulating specific acupoints, acupuncture can regulate the activity of immune cells in the gastrointestinal tract, reduce the levels of immune factors such as IL-1 and TNF-α, and alleviate sleep disturbances caused by immune responses, thereby helping to restore normal sleep patterns.

Jinfeng et al. (2008) and Yanli and Xianfeng (2012) demonstrated through animal experiments that acupuncture can increase the levels of IL-1β and TNF-α in the hypothalamus of insomnia rat models, confirming that acupuncture can participate in the sleep regulation process by modulating the immune system. Zhao et al. (2008) showed through animal experiments that electroacupuncture can influence the synthesis and release of IL-1β, IL-6, and TNF-α in the hypothalamus, thereby improving the sleep state of rats. Shuangshuang (2013) demonstrated through clinical trials that the mechanism by which acupuncture combined with massage improves sleep quality is related to increasing TNF-α levels and decreasing IL-6 levels. These studies suggest that acupuncture may treat insomnia by regulating immune factors and thereby influencing the immune pathways of the MGBA.

In conclusion, acupuncture, as a safe and effective treatment method, improves sleep quality by influencing MGBA communication through various pathways. In addition to regulating metabolites, neurotransmitters, immune function, and the autonomic nervous system, acupuncture can also promote blood circulation, improve cerebral blood flow, and increase oxygen supply to the brain, thereby enhancing sleep quality. Moreover, acupuncture can alleviate emotional issues such as anxiety and depression, reduce physical and mental stress, and help improve insomnia symptoms such as difficulty falling asleep and frequent waking.

### 4.3 Acupuncture intervention

**Figure 2:**
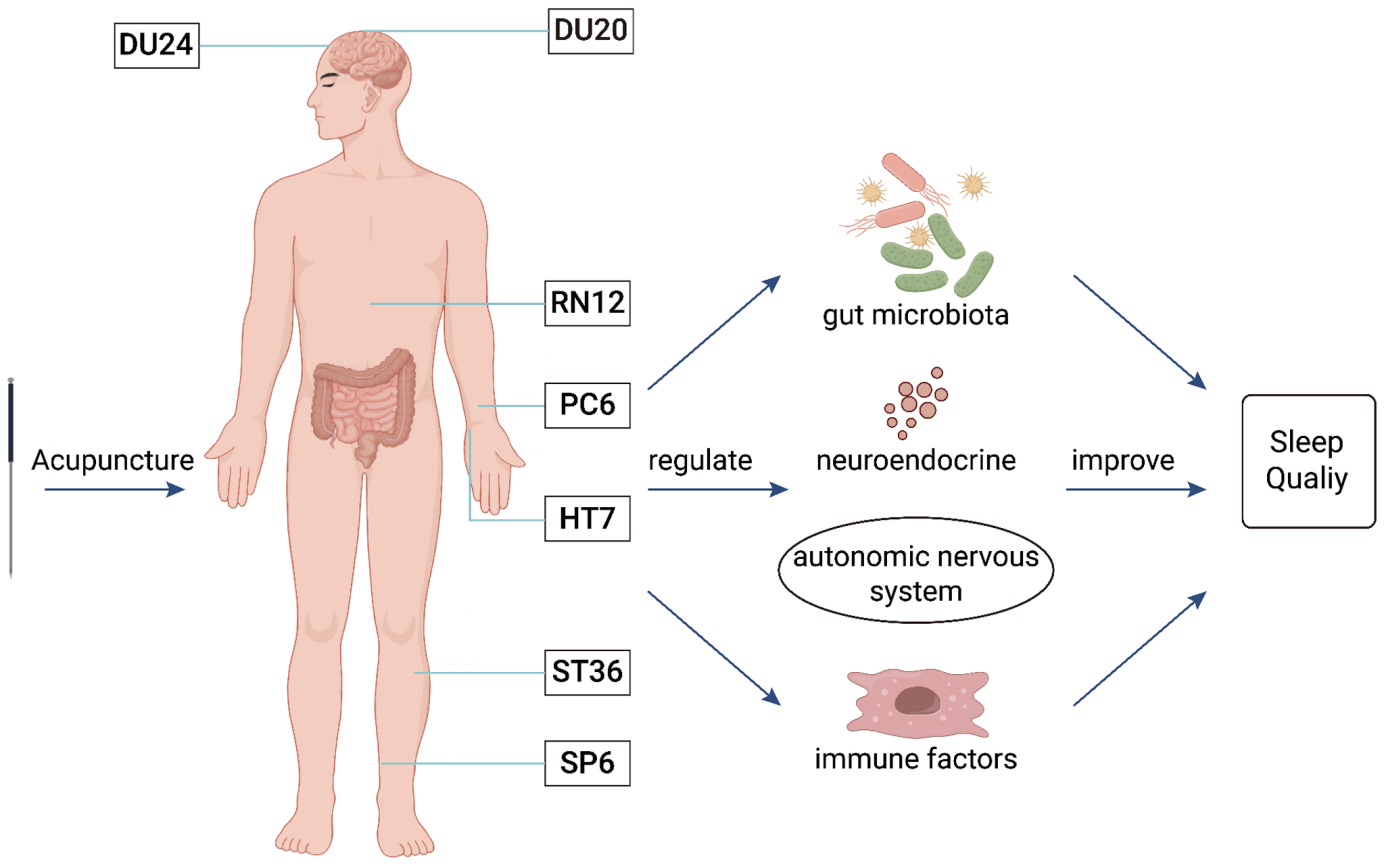
The mechanism of acupuncture intervention in the MGBA for the treatment of insomnia. This figure illustrates that acupuncture at Baihui (DU20), Shenting (DU24), Zhongwan (RN12), Neiguan (PC6), Shenmen (HT7), Zusanli (ST36), and Sanyinjiao (SP6) can regulate the gut microbiota, neurotransmitters, autonomic nervous system, and immune factors to improve insomnia.

In clinical practice, acupuncture has been widely used in the treatment of insomnia. However, there is no standardized protocol for acupuncture prescriptions for insomnia, and it remains in the exploratory stage of operational standardization. Based on the microbiota-gut-brain axis and existing evidence-based medicine (Liang et al., 2022), we believe that the primary acupuncture points for treating insomnia are Baihui (DU20), Shenting (DU24), Shenmen (HT7), Neiguan (PC6), Zhongwan (RN12), Zusanli (ST36), and Sanyinjiao (SP6) (Figure 2).

Baihui (DU20) is located on the head and belongs to the Du meridian. It is the point where all meridians converge, allowing the energy from various meridians to gather and permeate the entire body. Acupuncture at Baihui (DU20) has the effect of regulating the body’s yin-yang balance and improving brain function. Modern medicine suggests (Ran et al., 2019) that acupuncture at Baihui (DU20) can maintain the normal sleep–wake cycle rhythm by activating the hypothalamic region and increasing signals in the temporal lobe.

Shenting (DU24) is a meeting point of the Du meridian, Stomach meridian of Foot-Yangming, and Bladder meridian of Foot-Taiyang, where the yang energy of the Du meridian gathers. Acupuncture at Shenting (DU24) can regulate the inflow and outflow of yang energy, balancing it with yin energy. Experiments have shown (Yuanzheng et al., 2021) that electroacupuncture at the Baihui (DU20)–Shenting (DU24) point combination can alleviate autonomic nervous system dysfunction and regulate neurotransmitter levels, thereby improving insomnia.

Shenmen (HT7), the original point of the Heart meridian, serves as the portal for heart energy. Acupuncture at Shenmen (HT7) has the effect of calming the mind and nourishing heart energy. Research (Hui et al., 2011) found that acupuncture at Shenmen (HT7) can improve sleep by regulating the levels of 5-HT in the brain.

Zhongwan (RN12) is located on the abdomen and is the alarm point of the Stomach as well as the meeting point of the bowels, belonging to the Ren meridian. Acupuncture at Zhongwan (RN12) has the effect of harmonizing the stomach and treating all visceral diseases.

Zusanli (ST36) is the He-Sea point of the Stomach meridian of Foot-Yangming and the lower He-Sea point of the Stomach. Acupuncture at Zusanli (ST36) has the function of tonifying the spleen and harmonizing the stomach. Research by Peihong (2018) and others found that acupuncture at Zhongwan (RN12) and Zusanli (ST36) can significantly reduce plasma somatostatin levels and increase hypothalamic GABA levels, improving sleep disorders and thereby enhancing the quality of life for patients.

Neiguan (PC6) is the Luo-Connecting point of the Pericardium meridian. Ancient physicians believed that the pericardium could “act on behalf of the heart to receive pathogens” and is good at opening the chest and regulating qi. Acupuncture at Neiguan (PC6) can expel pathogens and regulate sleep. Fajun et al. (2020) and others confirmed that acupuncture at Zusanli (ST36), Neiguan (PC6), and Zhongwan (RN12) points can significantly improve serum 5-HT levels in insomnia patients, thereby improving sleep quality and maintaining long-term therapeutic effects.

Sanyinjiao (SP6) is the meeting point of the meridians of the Spleen, Kidney, and Liver. It can regulate the body’s yin and yang, allowing “yang to enter yin,” and improve insomnia symptoms by strengthening the spleen and stomach, regulating the liver, and nourishing the kidney. Research has found (Zhongwen et al., 2022) that acupuncture at Sanyinjiao (SP6) can increase the levels of serum GABA and 5-HT in the body, thereby improving sleep and enhancing sleep quality.

## 5 Discussion

From the above discussion, it is evident that insomnia is a complex sleep disorder involving dysregulation across multiple systems, including the nervous, endocrine, and immune systems. In recent years, the MGBA has emerged as a novel research field, providing new insights into the pathogenesis of insomnia. Acupuncture, as a traditional Chinese medical therapy, has demonstrated significant efficacy in the treatment of insomnia, and its mechanisms of action have gradually become a focal point of research. Acupuncture may treat insomnia by influencing the MGBA through the regulation of gut microbiota, neurotransmitters, certain hormones, the autonomic nervous system, and inflammatory factors. However, there are still many limitations and challenges at this stage. For instance, the connection between theory and clinical practice is not sufficiently strong. Acupuncture prescriptions for insomnia are varied and often depend on the clinical experience of practitioners, lacking experimental data to validate their scientific basis and feasibility. Additionally, while the feasibility of acupuncture for treating insomnia has been confirmed, the theoretical support for MGBA within traditional Chinese medicine is relatively weak, and there is limited research on the mechanisms by which acupuncture treats insomnia through the MGBA. Future research should further integrate basic science with clinical practice to explore the specific regulatory mechanisms of MGBA in insomnia and evaluate the effectiveness and safety of interventions such as acupuncture. This approach will not only deepen the understanding of the pathophysiological mechanisms of insomnia but also potentially lead to the development of more innovative and personalized treatment strategies to address this complex sleep disorder.

In summary, the MGBA offers a new perspective for comprehensively understanding the pathological mechanisms of insomnia, while also providing a scientific foundation for exploring the mechanisms of external therapies, such as acupuncture, in treating insomnia. Therefore, in-depth research on the relationship between MGBA and insomnia, as well as the specific mechanisms of acupuncture in the treatment of insomnia, holds significant practical importance for optimizing clinical treatment strategies. Although current research findings are limited, the field holds vast potential and is worthy of further exploration. With the continuous application of new technologies and theories, such as microbiomics and metagenomics, research on the relationship between the MGBA and insomnia is expected to deepen, leading to more comprehensive results. These advancements could provide innovative treatment methods for clinical practice and potentially open new avenues for the treatment of various diseases.

## References

[ref1] Asarnow L. D. (2019). Depression and sleep: what has the treatment research revealed and could the Hpa axis be a potential mechanism? Curr. Opin. Psychol. 34, 112–116. doi: 10.1016/j.copsyc.2019.12.002, PMID: 31962280 PMC8412030

[ref2] Bao C. Wu L. Wang D. Chen L. Jin X. Shi Y.. (2022). Acupuncture improves the symptoms, intestinal microbiota, and inflammation of patients with mild to moderate Crohn’s disease: a randomized controlled trial. EclinicalMedicine 45:101300. doi: 10.1016/j.eclinm.2022.101300, PMID: 35198926 PMC8850329

[ref3] Belkaid Y. Hand T. W. (2014). Role of the microbiota in immunity and inflammation. Cell 157, 121–141. doi: 10.1016/j.cell.2014.03.011, PMID: 24679531 PMC4056765

[ref4] Burgos A. P. Mao Y. K. Bienenstock J. Kunze W. A. (2014). The gut‐brain axis rewired: adding a functional vagal nicotinic "sensory synapse. FASEB J. 28, 3064–3074. doi: 10.1096/fj.13-245282 24719355

[ref5] Chen W. Chen Y. Aslam M. S. Shen J. Tong T. Yan S.. (2023). The effect of acupuncture on lateral habenular nucleus and intestinal microflora in depression model rats. Behav. Brain Res. 455:114627. doi: 10.1016/j.bbr.2023.114627 37619770

[ref6] Chen Z. Feng J. Hu S. Hua Y. Ma S. Fu W.. (2022a). *Bacillus subtilis* promotes the release of 5-Ht to regulate intestinal peristalsis in Stc mice via bile acid and its receptor Tgr5 pathway. Dig. Dis. Sci. 67, 4410–4421. doi: 10.1007/s10620-021-07308-4, PMID: 34797444

[ref7] Chen Z. Feng Y. Li S. Hua K. Fu S. Chen F.. (2022b). Altered functional connectivity strength in chronic insomnia associated with gut microbiota composition and sleep efficiency. Front. Psych. 13:1050403. doi: 10.3389/fpsyt.2022.1050403, PMID: 36483137 PMC9722753

[ref8] Chen Y. Xu J. Chen Y. (2021). Regulation of neurotransmitters by the gut microbiota and effects on cognition in neurological disorders. Nutrients 13:2099. doi: 10.3390/nu13062099, PMID: 34205336 PMC8234057

[ref9] Chinese Society Of Neurology and Sleep Disorders Group (2018). Guidelines for the diagnosis and treatment of insomnia in Chinese adults (2017 edition). Chin. J. Neurol. 51, 324–335. doi: 10.3760/cma.j.issn.1006-7876.2018.05.002

[ref10] Cristofori F. Dargenio V. N. Dargenio C. Miniello V. L. Barone M. Francavilla R. (2021). Anti-inflammatory and immunomodulatory effects of probiotics in gut inflammation: a door to the body. Front. Immunol. 12:578386. doi: 10.3389/fimmu.2021.578386, PMID: 33717063 PMC7953067

[ref11] Crumeyrolle-Arias M. Jaglin M. Bruneau A. Vancassel S. Cardona A. Dauge V.. (2014). Absence of the gut microbiota enhances anxiety-like behavior and neuroendocrine response to acute stress in rats. Psychoneuroendocrinology 42, 207–217. doi: 10.1016/j.psyneuen.2014.01.014, PMID: 24636517

[ref12] Doifode T. Giridharan V. V. Generoso J. S. Bhatti G. Barichello T. (2020). The impact of the microbiota-gut-brain axis on Alzheimer’s disease pathophysiology. Pharmacol. Res. 164:105314. doi: 10.1016/j.phrs.2020.105314 33246175

[ref13] Erny D. de Angelis A. L. H. Jaitin D. Wieghofer P. Staszewski O. David E.. (2015). Host microbiota constantly control maturation and function of microglia in the Cns. Nat. Neurosci. 18, 965–977. doi: 10.1038/nn.4030, PMID: 26030851 PMC5528863

[ref14] Fajun L. Huixing H. Jingbo Z. Miaomiao Y. Zhen W. (2020). Effects of acupuncture on serum brain-gut peptides and sleep quality in patients with primary insomnia of "disharmony of stomach qi" pattern. J. Integr. Trad. Chinese Western Med. Cardiovasc. Cerebrovasc. Dis. 18, 2042–2045. doi: 10.12102/j.issn.1672-1349.2020.13.006

[ref15] Fernandes P. A. C. M. (2006). Effect of Tnf-alpha on the melatonin synthetic pathway in the rat pineal gland: basis for a ‘feedback’ of the immune response on circadian timing. J. Pineal Res. 41, 344–350. doi: 10.1111/j.1600-079X.2006.00373.x, PMID: 17014691

[ref16] Fock E. Parnova R. (2023). Mechanisms of blood–brain barrier protection by microbiota-derived short-chain fatty acids. Cells 12:657. doi: 10.3390/cells12040657, PMID: 36831324 PMC9954192

[ref17] Ge T. Yao X. Zhao H. Yang W. Zou X. Peng F.. (2021). Gut microbiota and neuropsychiatric disorders: implications for neuroendocrine-immune regulation. Pharmacol. Res. 173:105909. doi: 10.1016/j.phrs.2021.105909 34543739

[ref18] Guo J. Wang L. P. Liu C. Z. Zhang J. Wang G. L. Yi J. H.. (2013). Efficacy of acupuncture for primary insomnia: a randomized controlled clinical trial. Evid. Based Complement. Alternat. Med. 2013:163850. doi: 10.1155/2013/163850, PMID: 24159338 PMC3789397

[ref19] Hang S. Paik D. Yao L. Kim E. Huh J. R. (2019). Bile acid metabolites control Th17 and Treg cell differentiation. Nature 576, 143–148. doi: 10.1038/s41586-019-1785-z, PMID: 31776512 PMC6949019

[ref20] Hansen M. Sickelmann F. Pietrowsky R. Fehm H. L. Born J. (1997). Subdiaphragmatic vagotomy blocks the sleep-and fever-promoting effects of interleukin-1…. Am. J. Physiol. 273, R548–R553. doi: 10.1152/ajpregu.1997.273.4.R1246, PMID: 9362287

[ref21] Hao X. Ding N. Zhang Y. Yang Y. Zhao Y. Zhao J.. (2022). Benign regulation of the gut microbiota: the possible mechanism through which the beneficial effects of manual acupuncture on cognitive ability and intestinal mucosal barrier function occur in app/Ps1 mice. Front. Neurosci. 16:960026. doi: 10.3389/fnins.2022.960026, PMID: 35992924 PMC9382294

[ref22] Hong J. Chen J. Kan J. Liu M. Yang D. (2020). Effects of acupuncture treatment in reducing sleep disorder and gut microbiota alterations in Pcpa-induced insomnia mice. Evid. Based Complement. Alternat. Med. 2020:3626120. doi: 10.1155/2020/3626120 33178314 PMC7647758

[ref23] Hui W. Xu W. Huiling T. Limin B. (2011). Effects of acupuncture at Shenmen and Mingmen points on serotonin and 5-hydroxyindoleacetic acid levels in the dorsal raphe nucleus of rats. J. Trad. Chinese Med. 52, 1038–1040. doi: 10.13288/j.11-2166/r.2011.12.016

[ref24] Ingiosi A. M. Raymond R. M. Jr. Pavlova M. N. Opp M. R. (2015). Selective contributions of neuronal and astroglial interleukin-1 receptor 1 to the regulation of sleep. Brain Behav. Immun. 48, 244–257. doi: 10.1016/j.bbi.2015.03.014, PMID: 25849975

[ref25] Jiao Y. Guo X. Luo M. Li S. Rong P. (2020). Corrigendum to "effect of transcutaneous vagus nerve stimulation at auricular concha for insomnia: a randomized clinical trial". Evid. Based Complement. Alternat. Med. 2020:2536573. doi: 10.1155/2020/2536573, PMID: 33335555 PMC7723490

[ref26] Jinfeng H. Chaohui W. Yanying Q. Dan S. (2008). Research on the regulation of brain cytokines in insomniac rats by the acupuncture method of ‘regulating the spirit of the five Zang organs through the five Zang Shu points’ and its mechanism. Jilin Trad. Chinese Med. 28, 688–689. doi: 10.13463/j.cnki.jlzyy.2008.09.030

[ref27] Jinpeng Z. Ying G. Bin Z. Huijie C. Shuai S. Luwen Z.. (2022). A randomized controlled study on the treatment of post-stroke insomnia with retained filiform fire needles at five Xin points combined with repetitive transcranial magnetic stimulation. J. Clin. Acupunct. 38:6. doi: 10.19917/j.cnki.1005-0779.022222

[ref28] Jones L. A. Sun E. W. Martin A. M. Keating D. J. (2020). The ever-changing roles of serotonin. Int. J. Biochem. Cell Biol. 125:105776. doi: 10.1016/j.biocel.2020.105776, PMID: 32479926

[ref29] Kim S. Lee S. Kim J. Van Den Noort M. Bosch P. Won T.. (2021). Efficacy of acupuncture for insomnia: a systematic review and meta-analysis. Am. J. Chin. Med. 49, 1135–1150. doi: 10.1142/S0192415X21500543 34049475

[ref30] Krueger J. M. Majde J. A. Rector D. M. (2011). Cytokines in immune function and sleep regulation. Handb. Clin. Neurol. 98, 229–240. doi: 10.1016/B978-0-444-52006-7.00015-0, PMID: 21056190 PMC5440845

[ref31] Kuwahara A. Matsuda K. Kuwahara Y. Asano S. Inui T. Marunaka Y. (2020). Microbiota-gut-brain axis: Enteroendocrine cells and the enteric nervous system form an interface between the microbiota and the central nervous system. Biomed. Res. 41, 199–216. doi: 10.2220/biomedres.41.199 33071256

[ref32] Lan Y. Lu J. Qiao G. Mao X. Zhao J. Wang G.. (2023). *Bifidobacterium breve* Ccfm1025 improves sleep quality via regulating the activity of the Hpa axis: a randomized clinical trial. Nutrients 15:4700. doi: 10.3390/nu15214700, PMID: 37960353 PMC10648101

[ref33] Legan T. B. Lavoie B. Mawe G. M. (2022). Direct and indirect mechanisms by which the gut microbiota influence host serotonin systems. Neurogastroenterol. Motility 34:e14346. doi: 10.1111/nmo.14346, PMID: 35246905 PMC9441471

[ref34] Lehahn Y. (2022). Sleep disorders and intestinal diseases: research progress based on gut microbiota. Clin. Med. Adv. 12, 6910–6916. doi: 10.12677/Acm.2022.127996

[ref35] Li Y. Li W. Wang S. Gong Y. Dou B. Lyu Z.. (2022). The autonomic nervous system: a potential link to the efficacy of acupuncture. Front. Neurosci. 16:1038945. doi: 10.3389/fnins.2022.1038945, PMID: 36570846 PMC9772996

[ref36] Li N. Tan S. Wang Y. Deng J. Wang N. Zhu S.. (2023). *Akkermansia muciniphila* supplementation prevents cognitive impairment in sleep-deprived mice by modulating microglial engulfment of synapses. Gut Microbes 15:2252764. doi: 10.1080/19490976.2023.2252764 37671803 PMC10484034

[ref37] Liang G. Yufeng B. Changchun J. Zhangyin S. Sirui X. (2022). A study on acupoint selection patterns for acupuncture treatment of primary insomnia. J. Liaoning Univ. Tradit. Chin. Med. 24, 104–108. doi: 10.13194/j.issn.1673-842x.2022.07.024

[ref38] Liu C. Yang M. Zhang G. Wang X. Li B. Li M.. (2020). Neural networks and the anti-inflammatory effect of transcutaneous auricular vagus nerve stimulation in depression. J. Neuroinflammation 17:54. doi: 10.1186/s12974-020-01732-5 32050990 PMC7017619

[ref39] Lucena L. D. R. Loyola V. T. Bortolli C. L. D. Andersen M. L. Tufik S. Hachul H. (2021). Effects of supplementation with Lactobacillus probiotics on insomnia treatment. Altern. Ther. Health Med. 27, 178–184. doi: 10.3410/f.1103972.561126 33609341

[ref40] Mabunga D. F. N. Gonzales E. L. T. Kim H. J. Choung S. Y. (2015). Treatment of Gaba from fermented rice germ ameliorates caffeine-induced sleep disturbance in mice. Biomol. Ther. 23, 268–274. doi: 10.4062/biomolther.2015.022, PMID: 25995826 PMC4428720

[ref41] Manosso L. M. Duarte L. A. Martinello N. S. Mathia G. B. Réus G. Z. (2024). Circadian rhythms and sleep disorders associated to major depressive disorder: pathophysiology and therapeutic opportunities. CNS Neurol. Disord. Drug Targets 23, 1085–1100. doi: 10.2174/0118715273254093231020052002, PMID: 37885113

[ref42] Morais L. H. Iv H. L. S. Mazmanian S. K. (2020). The gut microbiota–brain axis in behaviour and brain disorders. Nat. Rev. Microbiol. 19, 241–255. doi: 10.1038/s41579-020-00460-0 33093662

[ref43] Morin C. M. Jarrin D. C. (2022). Epidemiology of insomnia: prevalence, course, risk factors, and public health burden. Sleep Med. Clin. 17, 173–191. doi: 10.1016/j.jsmc.2022.03.003 35659072

[ref44] Mossad O. Erny D. (2020). The microbiota–microglia axis in central nervous system disorders. Brain Pathol. 30, 1159–1177. doi: 10.1111/bpa.12908, PMID: 33073887 PMC8018151

[ref45] Onyszkiewicz M. Gawry-Kopczyńska M. Konopelski P. Aleksandrowicz M. Ufnal M. (2019). Butyric acid, a gut bacteria metabolite, lowers arterial blood pressure via colon-vagus nerve signaling and Gpr41/43 receptors. Pflugers Arch. - Eur. J. Physiol. 471, 1441–1453. doi: 10.1007/s00424-019-02322-y, PMID: 31728701 PMC6882756

[ref46] Pandi-Perumal S. R. Trakht I. Spence D. W. Srinivasan V. Dagan Y. Cardinali D. P. (2008). The roles of melatonin and light in the pathophysiology and treatment of circadian rhythm sleep disorders. Nat. Clin. Pract. Neurol. 4, 436–447. doi: 10.1038/ncpneuro0847, PMID: 18628753

[ref47] Patterson E. Tan H. T. T. Groeger D. Andrews M. Buckley M. Murphy E. F.. (2024). Bifidobacterium longum 1714 improves sleep quality and aspects of well-being in healthy adults: a randomized, double-blind, placebo-controlled clinical trial. Sci. Rep. 14:3725. doi: 10.1038/s41598-024-53810-w, PMID: 38355674 PMC10866977

[ref48] Peihong M. (2018). A study on the effects of acupuncture with He-Mu acupoint combination on symptom improvement and plasma somatostatin levels in patients with functional dyspepsia. Chengdu: Chengdu University of Traditional Chinese Medicine.

[ref49] Poroyko V. A. Carreras A. Khalyfa A. Khalyfa A. A. Leone V. Peris E.. (2016). Chronic sleep disruption alters gut microbiota, induces systemic and adipose tissue inflammation and insulin resistance in mice. Sci. Rep. 6:35405. doi: 10.1038/srep35405, PMID: 27739530 PMC5064361

[ref50] Provided H. A. T. T. Zhong H. Shao X. Hu S. She C. Liu X.. (2019). Progress in research on the mechanisms of acupuncture based on metabolomics. World J. Trad. Chinese Med. 170, 116031–116547. doi: 10.1016/j.biopha.2023.116031

[ref51] Qiang L. Linlin D. Fei W. Lijuan W. Jinhua G. (2020). Clinical study on the treatment of insomnia with high-frequency electroacupuncture combined with the "Xiao Xingnao Kaiqiao" acupuncture method. J. Zhejiang Chinese Med. Univ. 43, 1284–1287. doi: CNKI:SUN:BHON.0.2019-11-021

[ref52] Qinghai Y. Rongyu L. (2006). Research progress on stress events and hypothalamic-pituitary-adrenal axis interaction in patients with obstructive sleep apnea-hypopnea syndrome. Int. J. Respir. 26, 438–440. doi: 10.3760/cma.j.issn.1673-436X.2006.06.012

[ref53] Qiu X. Li Z. Huang S. Cai X. Qu S. Zheng Z.. (2023). Electroacupuncture improves depression-like behavior by regulating the abundance of Lactobacillus and staphylococci in mice. J. Integr. Neurosci. 22:28. doi: 10.31083/j.jin2202028 36992578

[ref54] Ran M. Lihong K. Fengjun Q. Feng S. Wei M. (2019). An analysis of ancient and modern research on the effects of Baihui acupoint on the brain. Liaoning J. Trad. Chinese Med. 46, 425–428. doi: 10.13192/j.issn.1000-1719.2019.02.065

[ref55] Rancillac A. (2016). Serotonin and sleep-promoting neurons. Oncotarget 7, 78222–78223. doi: 10.18632/oncotarget.13419, PMID: 27861160 PMC5346632

[ref56] Rashid I. Mir M. A. Andleeb A. Kumar A. Munshi U. Habib D.. (2024). Role of melatonin receptors as regulators of neurophysiology and therapeutic targets. J. Pharma Insights Res. 2, 255–265. doi: 10.5281/zenodo.11200118

[ref57] Rong P. Liu J. Wang L. Liu R. Fang J. Zhao J.. (2016). Effect of transcutaneous auricular vagus nerve stimulation on major depressive disorder: a nonrandomized controlled pilot study. J. Affect. Disord. 195, 172–179. doi: 10.1016/j.jad.2016.02.031 26896810 PMC4828906

[ref58] Rothhammer V. Mascanfroni I. D. Bunse L. Takenaka M. C. Kenison J. E. Mayo L.. (2016). Type I interferons and microbial metabolites of tryptophan modulate astrocyte activity and central nervous system inflammation via the aryl hydrocarbon receptor. Nat. Med. 22, 586–597. doi: 10.1038/nm.4106, PMID: 27158906 PMC4899206

[ref59] Shuangshuang Z. (2013). Effects of acupuncture combined with Tuina on Tnf-α and Il-6 in patients with heart-spleen qi deficiency type insomnia. Hubei: Hubei University of Traditional Chinese Medicine.

[ref60] Silva Y. P. Bernardi A. Frozza R. L. (2020). The role of short-chain fatty acids from gut microbiota in gut-brain communication. Front. Endocrinol. 11:25. doi: 10.3389/fendo.2020.00025, PMID: 32082260 PMC7005631

[ref61] Strandwitz P. Kim K. H. Terekhova D. Liu J. K. Sharma A. Levering J.. (2019). Gaba-modulating bacteria of the human gut microbiota. Nat. Microbiol. 4, 396–403. doi: 10.1038/S41564-018-0307-3 30531975 PMC6384127

[ref62] Sudo N. Chida Y. Aiba Y. Sonoda J. Oyama N. Yu X. N.. (2004). Postnatal microbial colonization programs the hypothalamic-pituitary-adrenal system for stress response in mice. J. Physiol. 558, 263–275. doi: 10.1113/jphysiol.2004.063388, PMID: 15133062 PMC1664925

[ref63] Szentirmai É. Krueger J. M. (2014). Sickness behaviour after lipopolysaccharide treatment in ghrelin deficient mice. Brain Behav. Immunity 36, 200–206. doi: 10.1016/j.bbi.2013.11.017, PMID: 24309634 PMC3951816

[ref64] Szentirmai É. Millican N. S. Massie A. R. Kapás L. (2019). Butyrate, a metabolite of intestinal bacteria, enhances sleep. Sci. Rep. 9:7035. doi: 10.1038/s41598-019-43502-1, PMID: 31065013 PMC6504874

[ref65] Tianyue Y. Yunfang H. Xue W. Ziqi J. Yujie Z. Abelson H. (2023). Comparative study on bile acid composition and content difference between two kinds of insomnia mouse models. China J. Trad. Chinese Med. Pharm. 38, 4346–4351.

[ref66] Van de Wouw M. Boehme M. Lyte J. M. Wiley N. Strain C. O'sullivan O.. (2018). Short-chain fatty acids: microbial metabolites that alleviate stress-induced brain-gut axis alterations. J. Physiol. 596, 4923–4944. doi: 10.1113/Jp276431, PMID: 30066368 PMC6187046

[ref67] Wang Z. Chen W. Li S. He Z. Zhu W. Ji Y.. (2021). Gut microbiota modulates the inflammatory response and cognitive impairment induced by sleep deprivation. Mol. Psychiatry 26, 6277–6292. doi: 10.1038/s41380-021-01113-1, PMID: 33963281

[ref68] Wang X. Wang Z. Cao J. Dong Y. Chen Y. (2023). Gut microbiota-derived metabolites mediate the neuroprotective effect of melatonin in cognitive impairment induced by sleep deprivation. Microbiome 11:17. doi: 10.1186/s40168-022-01452-3, PMID: 36721179 PMC9887785

[ref69] Wei G. Z. Martin K. A. Xing P. Y. Agrawal R. Whiley L. Wood T. K.. (2021). Tryptophan-metabolizing gut microbes regulate adult neurogenesis via the aryl hydrocarbon receptor. Proc. Natl. Acad. Sci. 118:e2021091118. doi: 10.1073/pnas.2021091118, PMID: 34210797 PMC8271728

[ref70] Xian W. Aihua L. (2015). Mechanisms of vagus nerve stimulation in regulating sleep: research advances. J. Stroke Neurol. Disord. 32, 1140–1141.

[ref71] Xu M. M. Guo Y. Chen Y. Zhang W. Wang L. Li Y. (2023). Electro-acupuncture promotes gut motility and alleviates functional constipation by regulating gut microbiota and increasing butyric acid generation in mice. J. Integr. Med. 21, 397–406. doi: 10.1016/j.joim.2023.05.003, PMID: 37331860

[ref72] Xu H. Wu L. Zhang Y. Ba T. Zhao X. (2024). Efficacy and safety of electroacupuncture for insomnia: a systematic review and meta-analysis. J. Integr. Med. 22, 459–472. doi: 10.1016/j.joim.2024.05.005 38871592

[ref73] Yanli Z. Xianfeng Y. (2012). Experimental study on the effects of acupuncture at different points on the levels of Il-1 and Tnf-α in the brain of insomnia model rats. Chinese J. Basic Med. Trad. Chinese Med. 18, 419–420. doi: 10.19945/j.cnki.issn.1006-3250.2012.04.033

[ref74] Yanlong H. (2004). Study on the effects and mechanisms of lateral ventricle injection of substance P on sleep-wake cycles in rats. Chinese Pharmacol. Bull. 20:2. doi: 10.3321/j.issn:1001-1978.2004.11.033

[ref75] Yi T. Zheng Q. Huang J. Ying C. (2023). Effect of acupuncture on the endocrine axis in patients with perimenopausal insomnia: a case series study. World J. Acupunct. Moxibust. 33, 97–101. doi: 10.1016/j.wjam.2022.05.007

[ref76] Yichuang H. (2023). A systematic review of acupuncture for the treatment of insomnia and a study on acupoint selection patterns. Heilongjiang: Heilongjiang University of Chinese Medicine.

[ref77] Yu L. Han X. Cen S. Duan H. Feng S. Xue Y.. (2020). Beneficial effect of Gaba-rich fermented milk on insomnia involving regulation of gut microbiota. Microbiol. Res. 233:126409. doi: 10.1016/j.micres.2020.126409, PMID: 31927503

[ref78] Yuanzheng S. Yue L. Tianyang Y. (2021). Effect of regulating Shen electrostimulation combined with repetitive transcranial magnetic stimulation on sleep disorders in stroke patients with heart-spleen qi deficiency and its impact on Hrv, 5-Ht, and ne. Shizhen Natl. Med. Natl. Pharm. 32, 2699–2702.

[ref79] Zang S. Chen Y. Chen H. Shi H. Zhou L. (2023). Effects of acupuncture on the brain in primary insomnia: a coordinate-based meta-analysis of fmri studies. Front. Neurol. 14:1180393. doi: 10.3389/fneur.2023.1180393, PMID: 37533466 PMC10392941

[ref80] Zengjie Y. Muzi L. Qu H. Yuanliang Y. Shuni W. Hongzhong Q. (2017). Advances in research on insomnia disorders at home and abroad. Med. Philos. B 38:4. doi: 10.12014/j.issn.1002-0772.2017.05b.17

[ref81] Zhang Z. Zhang Y. Li J. Fu C. Zhang X. (2021). The neuroprotective effect of tea polyphenols on the regulation of intestinal flora. Molecules 26:3692. doi: 10.3390/molecules26123692, PMID: 34204244 PMC8233780

[ref82] Zhao C. Ren L. Song Y. (2008). The effects of electroacupuncture at different acupoint prescriptions on hypothalamic Il-1β, Tnf-α, and Il-6 in insomnia rats. J. Jinan Univ. Nat. Sci. Med. Ed. 29, 177–179. doi: 10.3969/j.issn.1000-9965.2008.02.017

[ref83] Zhongwen L. Ling Y. Xiaojun S. Du L. Yihui Z. (2022). Effects of Shenmen and Sanyinjiao combination on sleep quality and serum Gaba, 5-Ht in insomnia. World Sci. Technol. Modern. Trad. Chinese Med. 24, 860–866.

[ref84] Zhou R. Qian S. Cho W. C. Zhou J. Jin C. Zhong Y.. (2022). Microbiota‐microglia connections in age‐related cognition decline. Aging Cell 21:e13599. doi: 10.1111/acel.13599 35349746 PMC9124309

[ref85] Zielinski M. Gibbons A. J. (2022). Neuroinflammation, sleep, and circadian rhythms. Front. Cell. Infect. Microbiol. 12:853096. doi: 10.3389/fcimb.2022.853096, PMID: 35392608 PMC8981587

[ref86] Zielinski M. R. McKenna J. T. McCarley R. W. (2016). Functions and mechanisms of sleep. Aims Neurosci. 3, 67–104. doi: 10.3934/Neuroscience.2016.1.67, PMID: 28413828 PMC5390528

